# Cajanin Stilbene Acid Inhibited Vancomycin-Resistant *Enterococcus* by Inhibiting Phosphotransferase System

**DOI:** 10.3389/fphar.2020.00473

**Published:** 2020-04-15

**Authors:** Shengnan Tan, Xin Hua, Zheyong Xue, Jianzhang Ma

**Affiliations:** ^1^ Key Laboratory of Saline-alkali Vegetation Ecology Restoration (Northeast Forestry University), Ministry of Education, Harbin, China; ^2^ College of Pharmacy, Zhejiang Chinese Medical University, Hangzhou, China; ^3^ College of Wildlife and Protected Area, Northeast Forestry University, Harbin, China; ^4^ College of Life Science, Northeast Forestry University, Harbin, China

**Keywords:** vancomycin-resistant *Enterococcus* (VRE), phosphotransferase system (PTS), cajanin stilbene acid (CSA), proteomics, inhibition mechanism

## Abstract

Antimicrobial resistance has become a serious threat to human and animal health, and vancomycin-resistant *Enterococcus* has become an important nosocomial infection pathogen, causing thousands of deaths each year. In this study, after screening a variety of natural products, we found that cajanin stilbene acid (CSA) had significant inhibitory effect on sensitive and vancomycin-resistant *Enterococcus* (VRE) *in vitro*. And we also confirmed that CSA had significant anti-VRE infection ability *in vivo*. Subsequently, we studied the antibacterial mechanism of CSA through proteomics experiments, and the results showed that CSA killed *Enterococcus* by inhibiting the phosphotransferase system of *Enterococcus*, thus hinders the normal growth and metabolic functions of bacteria. The results of this study provided evidence for the in-depth study on the mechanism of the antibacterial action of CSA and also provided a candidate for the development of anti-VRE drugs.

## Introduction

*Enterococcus* is one of the most common conditionally pathogenic gram-positive bacteria, when the human body immunity is low or defects, *Enterococcus* will lead to multiple organ infection, such as urinary tract infection, soft tissue infection, it also cause severe infection such as bacteremia and endocarditis (Arias and Murray, 2012; Van Harten et al., 2017). *Enterococcus* is considered as the important nosocomial infection pathogens second only to *Staphylococcus aureus* (Cattoir and Giard, 2014). Vancomycin, a glycopeptide antibiotic, is used as a “last line of defense” against severe *enterococcal* and other Gram-positive bacterial infections which do not respond to other antibiotics (Park and Liu, 2012). In 2017, according to “Antibiotic resistance threats in the United States” (www.cdc.gov/DrugResistance/Biggest-Threats.html), about 54,500 people were infected with vancomycin-resistant *Enterococcus* (VRE) and 5,400 people died of VRE infection in the United States. Therefore, it is of great significance to develop new anti-VRE drug candidates.

Plants contain a considerable number of natural products with medicinal activity, which have been paid more and more attention in anti-infection, and are a promising source of new chemical entities with development potential (Thomford et al., 2018). At present, natural products from a variety of plant sources have been assigned with significant antibacterial activity (Silva et al., 2016; Salam and Quave, 2018). In order to search for new anti-VRE natural products, more than 100 natural products were screened for anti-VRE activities. We found cajanin stilbene acid (CSA) isolated from leaves of *Cajanus cajan* was found to be with significant anti-VRE activity. CSA showed to have antioxidant and antimicrobial activities as well as cytotoxicity toward cancer cells in previous reports (Zu et al., 2010; Wu et al., 2011; Fu et al., 2015; Huang et al., 2016). In this study, we found for the first time that CSA could significantly inhibit VRE *in vitro* and *in vivo*. Through the study, we also found that CSA inhibited the phosphotransferase system of VRE, and then inhibited the normal growth and metabolism of bacteria, thus achieving antibacterial activity. This study elucidated the unique antibacterial mechanism of CSA and provided evidence for the development of VRE antibacterial drug candidates.

## Method

### Strains, Cell Lines, and Antimicrobial Agents

In this experiment, the standard strains of *Enterococcus* and VRE were selected as *Enterococcus faecalis* ATCC29212 and vancomycin—resistant *E. faecalis* ATCC700802/V583, respectively. The detailed information of clinical strains is shown in **Table S1**. All strains involved in this experiment were presented by the First Affiliated Hospital of Harbin Medical University, Harbin, China. All strains were cultured in either Mueller-Hinton agar (MHA) or Mueller-Hinton broth (MHB). Mice macrophage RAW 264.7 were cultured in Dulbecco’s modified eagle medium (DMEM) (Invitrogen, both, MD, USA) supplemented with 10% fetal bovine serum (Gibco, Grand Island, NY) at 37°C. CSA was separated by our laboratory, purity > 98%, dissolved in DMSO (Kong et al., 2009). Vancomycin was purchased from Sigma Aldrich (Bornem, Belgium).

### Minimum Inhibitory Concentration Determination

The strains to be detected were inoculated on MHA medium, cultured at 37°C for 16~24 h, and single colonies were selected to be inoculated on MHB medium. Then, the culture medium was diluted to a bacterial suspension of 1 × 10^5^ colony-forming unit (CFU)/ml in a 200 rpm shaker at 37°C for 16~24 h, based on the Clinical and Laboratory Standards Institute (CLSI) guidelines, the minimum inhibitory concentration (MIC) of CSA was determined by microbroth dilution method (**Table S1**).

### Time-Dependent Killing

V583 strains cultured overnight were diluted in MHB medium at 1:5,000. After 2 h at 37°C and 200 rpm, the strain was treated with 10 μg/ml (5 × MIC) vancomycin or CSA for 24 h, CFU was calculated and the experiment was repeated for three times.

### Scanning Electron Microscope Observation

The overnight culture medium of V583 strains were diluted to 1 × 10^5^ CFU/ml and treated with CSA for 4 h. SEM specimens were prepared as previously reported (Hua et al., 2018), and then sputter coated with gold for observation using a JSM 7500 (JEOL, Tokyo, Japan).

### Adhesion Experiment

V583 strains were inoculated in MHB medium containing 0.25, 0.5, and 1 g/ml CSA, respectively, and then diluted to 1 × 10^9^ CFU/ml after culture at 37°C to logarithmic growth stage. HeLa cells (1 × 10^6^ cells) in the 24-well cell culture plate were washed with PBS, and 1 ml bacterial suspension was added. The cells were cultured in 5% CO_2_ at 37°C for 1.5 h, then washed with PBS for six times. PBS solution containing 1% Triton X-100 was added and incubated for 10 min. The suspension was sucked out, diluted, and placed on MHA medium for overnight culture. The CFU was counted and the adhesion rate was calculated.

### *In Vivo* Antibacterial Experiment

*In vivo* antibacterial experiments were performed on BALB/c mice aged 6 weeks. Animal experiments are conducted in accordance with animal ethical guidelines and approved protocols. The animal experiments were approved by the Animal Ethics Committee of the Harbin Veterinary Research Institute of the Chinese Academy of Agricultural Sciences (approval number IACUC-2018-101).

In the lethal protection experiment, the mice were divided into six groups: control, 5% dimethyl sulfoxide (DMSO), 1 mg/kg vancomycin, 5 mg/kg vancomycin, 1 mg/kg CSA, and 5 mg/kg CSA group, 10 mice in each group were intraperitoneally injected with V583 strains 5.3 × 10^8^ CFU, and the mice were injected with the antimicrobials through the tail vein for 7 days after inoculation. The survival rate of each group was calculated after 7 days.

In the systemic non-lethal infection, the mice were divided into four groups: control, 5% DMSO, vancomycin (5 mg/kg), and CSA (5 mg/kg) group, 10 mice in each group were intraperitoneally injected with V583 strains 3.6 × 10^7^ CFU. The mice were inoculated with antimicrobials through the tail vein for 7 days. Organs (including the small intestine, spleen, and liver) were collected on day 1 and day 7, after ground in PBS, diluted, and cultured on MHA medium overnight, CFU was counted and the number of microbiomes was calculated, and pathological examination of the small intestine was performed.

### Proteomics Assay

Proteomics assay was used to compare the protein expression of V583 strain in CSA treated group and untreated group. The V583 strains were cultured overnight and diluted to 1 × 10^5^ CFU/ml. The treatment group was treated with 1 mg/ml CSA for 1 hour, centrifuged at 3,000 g for 10 min, washed with PBS for three times and then placed in liquid nitrogen. The subsequent protein extraction, proteomics determination, and data analysis were completed by PTM Biolabs Inc., Hangzhou, China. The mass spectrometry proteomics data have been deposited to the ProteomeXchange Consortium *via* the PRIDE partner repository with the dataset identifier PXD017522.

### Fluorescent Quantitative PCR

Total RNA was extracted by RNA extraction kit (Qiagen, Valencia, United State), and total RNA reverse transcription was performed by reverse transcription kit (Takara, Clontech, Japan). Real-time quantitative PCR primers were designed using primer premier 5.0, primer sequence is shown in **Table S2**. Then, SYBR GREEN was used for real time (RT)-quantitative PCR (PCR) identification. The reaction conditions were 95°C denaturation for 5 min, 95°C 40 cycles for 15 s, 55°C annealing for 15 s, 72°C extension for 15 s, finally, the 95°C fully extended 1 min, the expression of gene was calculated by 2^−Δ^ ^Ct^ method.

### Statistical Analysis

Statistical analyses were performed using GraphPad Prism 7.0 (GraphPad Software, La Jolla, CA). One-way ANOVA was performed between groups. For ANOVA, the observed variance is partitioned into components according to different explanatory variables. ***P < 0.005 was considered to be significant.

## Results and Discussion

**Figure 1:**
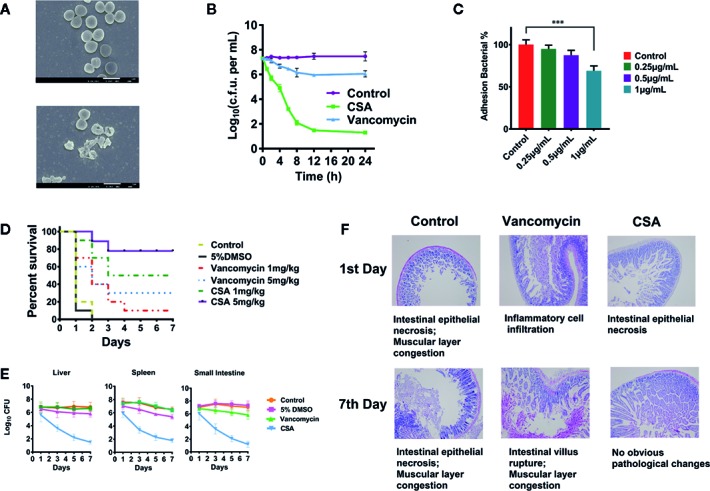
Inhibition of vancomycin-resistant *Enterococcus* (VRE) by cajanin stilbene acid (CSA) *in vitro* and *in vivo*. **(A)** SEM results of V583 strain treated with CSA, magnify by 20,000 times. **(B)** Results of time killing curve of V583 strain treated with 10 μg/ml CSA. **(C)** Survival rate of V583 infected mice treated with CSA. In systemic lethal infection, mice were intraperitoneally injected with 1.2 × 10^8^ colony-forming unit (CFU) of V583. ***The mice were then divided into six groups (10 mice per group) and tail vein injected with CSA, vancomycin, 5% dimethyl sulfoxide (DMSO), and normal saline, mortality was monitored daily for 7 days. **(D, E)** After CSA treatment, the bacterial load in liver, spleen, and small intestine of V583 infected mice and the pathological results. In systemic lethal infection, mice were intraperitoneally injected with 1.2 × 10^8^ CFU of V583. The mice were then divided into four groups (10 mice per group) and tail vein injected with CSA (5 mg/kg), vancomycin (5 mg/kg), 5% DMSO, and normal saline for 7 days. **(F)** Pathological section of the small intestine.

We determined the MIC of CSA against 40 strains of *Enterococcus*, including *E. faecalis* ATCC29212 and vancomycin—resistant *E. faecalis* ATCC700802/V583. The results showed that the CSA could effectively inhibit sensitive *Enterococcus* strains and VRE strains with MIC values between 0.5 and 2 μg/ml. The V583 strains and other clinical drug resistant strains showed lower sensitivity to vancomycin, whose MIC value ranged at 16–128 μg/ml (**Table S1**). After that, V583 strains was selected for the determination of the CSA bactericidal curve, and the strain was treated with 10 μg/ml (5 × MIC) vancomycin or CSA for 24 h. The results showed that CSA could kill a large proportion of bacterial cells in 4 h, kill 95% in 8 h, and finally reduce 3-log10 bacterial cells in 24 h (**Figure 1B**).

SEM was used to observe V583 strains morphology and survival state treated with 2 μg/ml of CSA after 4 h (**Figure 1A**). In the control group, the V583 strains had complete structure, smooth surface, and even cytoplasm, while the death of V583 could be obviously found after treated with CSA. After CSA treatment, the rupture of V583 cells and outflow of contents were observed, indicating that CSA was an antibacterial drug that could cause death of VRE.

In the experiment of inhibiting adhesion, CSA showed a strong anti-adhesion effect in dose-dependent pattern. After treated with 1 µg/ml CSA, the adhesion ability of V583 decreased by 30% (*P* < 0.005) (**Figure 1C**).

*In vivo* experiments, lethal model and semi-lethal model were both conducted. Rats were intraperitoneally injected with 5 × 10^8^ CFU of V583 strains. CSA and vancomycin were injected respectively with *via* tail intravenous twice a day, and the survival rate was calculated after 7 days. Mice in the control group and the solvent control group all died. When the dosages of 1 and 5 mg/kg were administered in CSA group, the survival rates were 50 and 90%, respectively (**Figure 1D**). When the dosages of 1 and 5 mg/kg were administered in vancomycin group, the survival rates were 10 and 30%, respectively. The experimental results showed that vancomycin had weak protection against VRE strains, while CSA had a significant protective effect on VRE infection in mice at both of high and low concentrations, with a significant dose-effect relationship.

In the semi-lethal trial, after injection of 1.2 × 10^7^ CFU of V5831, CSA, and vancomycin (5 mg/kg) were administered twice daily. We then measured the levels of the bacteria in the liver, spleen, and small intestine of the mice. After 7 days of administration, no significant changes were observed in the number of bacteria in all organs of the vancomycin group. In the group of CSA, the bacterial load in liver, spleen, and small intestinal were decreased from 3.2 × l0^7^ CFU to 8.9 × l0^2^ CFU, 2.4 × l0^7^ CFU to 2.1 × l0^3^ CFU and 9.5 × l0^6^ CFU to 6.8 × l0^2^ CFU, respectively (**Figure 1E**). The results showed that CSA significantly reduced the number of bacteria in all organs of VRE infected mice. Moreover, the section observation showed that the pathological changes of small intestine in CSA group were significantly reduced at 7 days compared with the control and vancomycin groups, which caused intestinal epithelial necrosis, intestinal villus rupture, and muscular layer congestion (**Figure 1F**).

The above results showed that CSA could significantly inhibit the activity of VRE strains *in vivo* and *in vitro*. Moreover, it was also known from previous reports that CSA had very low toxicity and could be used as a candidate drug to inhibit VRE strains (Fu et al., 2015).

In order to explore the molecular mechanism of CSA inhibiting VRE strain, we made a comparison between the proteomic data of *enterococcus* treated with or without CSA (1 μg/ml, 1 h). A total of 1,563 proteins were identified, of which 1,457 contained quantitative information. Two times was taken as the change threshold, and *t-test p*-value < 0.05 was considered as the standard. Comparative analysis of differences showed 90 up-regulated and 150 down-regulated proteins after CSA.

We also conducted a systematic bioinformatics analysis of proteins containing quantitative information, including protein annotation, functional classification, functional enrichment, and functional enrichment based cluster analysis. In order to show changes of protein expression level in more detail pattern, we sorted all differentially expressed proteins into four groups, named Q1 to Q4: Q1 (0 < ratio ≤ 1/2), Q2 (1/2 < ratio ≤ 1/1.5), Q3 (1.5 < ratio ≤ 2), and Q4 (ratio > 2). When the ratio is closer to 0, it means that the expression of the protein is significantly down-regulated after CSA treatment; when the ratio value is higher, the protein is more significantly up-regulated after CSA treatment. Then, Gene Ontology (GO) annotations and Kyoto Encyclopedia of Genes and Genomes (KEGG) pathways was performed for each Q group, and cluster analysis was conducted to find the correlation of protein functions with differentially expressed multiples.

**Figure 2:**
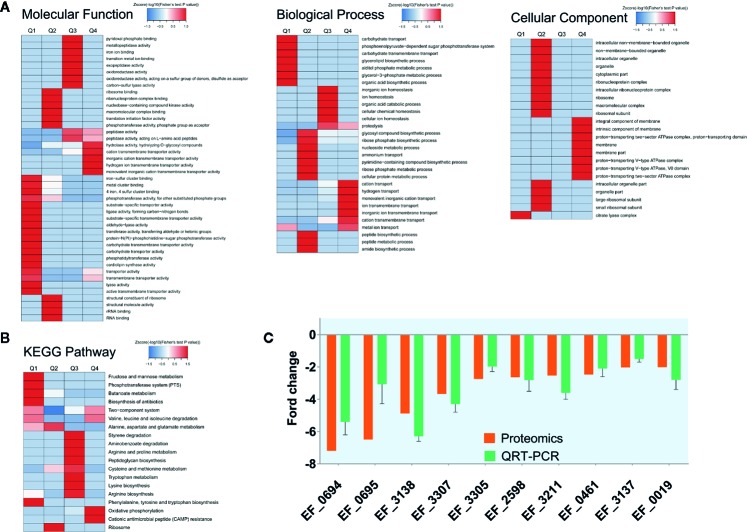
Protein expression results for V583 cells treated and not treated with cajanin stilbene acid (CSA). **(A)** Heatmap of cluster analysis based on Gene Ontology (GO) enrichment. There are three categories of GO, including biological process, cellular component, and molecular function. **(B)** Heatmap of cluster analysis based on Kyoto Encyclopedia of Genes and Genomes (KEGG) pathway. **(C)** Validation of proteomics data for selected genes by real-time PCR. The orange bar represents the proteomics results, while the green bar represents the quantitative real-time (QRT)-PCR results, experiments were performed with three replicates and data were showed in mean ± SD.

The GO-enriched cluster analysis included three categories: biological process, cellular component, and molecular function (**Figure 2A**). In the clustering of cell components, the genes involved in the citric acid lysis complex was significantly down-regulated, and the genes that were significantly up-regulated were mainly concentrated in the components of cell membrane and protein transport ATPase complex. In terms of molecular function, the downregulation mainly occurred in the metal ion binding, phosphorylase activity and carbohydrate transfer capacity. The upregulation mainly occurred in the hydrolase activity, cationic transmembrane transport activity, inorganic cationic transmembrane transport protein activity. In the biological process, the main downregulation was related to the carbohydrate transfer process and the carbohydrate transmembrane transport and other biological processes, while the up regulation was related to the cation transport process, hydrogen transport process, ion transmembrane transport, and other processes. From data of GO analysis in the cellular composition, molecular function, and biological process, we can find that the phosphorylase system of V583 strains and its related carbohydrate transport process are significantly inhibited, and some other transport-related proteins have also undergone significant changes after CSA treatment.

Similarly, in KEGG enriched cluster analysis, butanoate metabolism, phosphotransferase (PTS) system, fructose and mannose metabolic and biosynthesis of biosynthesis pathways were all significantly inhibited (**Figure 2B**). The fructose and mannose metabolic pathway were also likely to be down-regulated by PTS system inhibition.

PTS system are widespread in bacteria, playing important roles in a catalytic carbohydrate concomitant uptake and phosphorylation, it is also involved in carbohydrate transport, signal transduction, and catabolite repression (Opsata et al., 2010). PTS system ensures the efficient utilization of carbohydrate bacteria in a complex environment, and the steady growth and reproduction of bacteria (Rodionova et al.,2017). Previous reports suggested that PTS may be an important drug target (Huang et al., 2013). Disruption of the mannitol PTS (MPT operons) in *E. faecalis* resulted in extensive changes in the control of carbon catabolism and thus led to the imbalance of carbohydrate metabolism. Researchers found that anthraquinones and related compounds could effectively inhibit the EI part of PTS system and thus inhibit the growth of *E. faecalis* (Snyder et al., 2014).

Therefore, we suspected that CSA inhibited the PTS system, leading to the transport of carbohydrates by *Enterococcus*, and then destroyed the energy metabolism pathway of *Enterococcus*, so as to play an antibacterial effect. A total of 35 PTS proteins were quantified in our proteome data. Ten PTS proteins had two-fold decreased expression level. Among which nine proteins are carbohydrate specific type II transporters, including the specific transporters of mannose and sorbitol (**Table S3**). According to the results of proteomics, it is speculated that CSA might influence transport of carbohydrates by inhibiting the PTS system, thereby disrupting the energy metabolism pathway in *Enterococcus*, thus showing an antibacterial effect. This result was also exactly consistent with the analysis results of GO. The KEGG enriched cluster analysis also showed phosphotransferase (PTS) in the fructose and mannose metabolic pathways were significantly inhibited (**Figure 2B**), reducing the transport capacity of fructose and mannose-related proteins.

Then, fluorescence quantitative PCR was used to determine the expression level of the coding genes of the significantly down-regulated PTS proteins. After CSA treatment, the coding genes of the PTS proteins were significantly down-regulated, which was consistent with the results of protein sequencing (**Figure 2C**).

## Conclusion

Here we discovery an active natural products CSA which having a significant inhibition of VRE. Using proteomics and fluorescence quantitative PCR, we found CSA can inhibit carbohydrate specificity of the PTS system of type II transporters, thereby inhibiting the *Enterococcus* transshipment and intake of carbohydrates, eventually lead to abnormal energy metabolism and death.

## Data Availability Statement

The mass spectrometry proteomics data have been deposited to the ProteomeXchange Consortium *via* the PRIDE partner repository with the dataset identifier PXD017522.

## Ethics Statement

The animal study was reviewed and approved by Animal Ethics Committee of the Harbin Veterinary Research Institute of the Chinese Academy of Agricultural Sciences.

## Author Contributions

XH and ZX designed research. XH and ST analyzed data. ST performed research. JM wrote the paper.

## Funding

Supported by Opening Project of Zhejiang Provincial Preponderant and Characteristic Subject of Key University (Traditional Chinese Pharmacology), Zhejiang Chinese Medical University (No. ZYAOX2018012).

## Conflict of Interest

The authors declare that the research was conducted in the absence of any commercial or financial relationships that could be construed as a potential conflict of interest.
