# Supplementary material for: Cajanin Stilbene Acid Inhibited Vancomycin-Resistant Enterococcus by Inhibiting Phosphotransferase System
Source: Front Pharmacol. 2020 Apr 15;11:473. doi: 10.3389/fphar.2020.00473 (PMC7179074; doi:10.3389/fphar.2020.00473)
Supplement: Supplementary file 1 [file DataSheet_1.docx]

| Strains | Description | CSA | Vancomycin |
| --- | --- | --- | --- |
|  |  | MIC (μg/mL) | |
| ATCC29212 | *Enterococcus faecium* strain, purchased from ATCC | 1 | 0.25 |
| SC1 | *Enterococcus faecalis* strain, human stool isolate, 2012 | 1 | 0.25 |
| SC2 | *Enterococcus faecium* strain, human stool isolate, 2012 | 2 | 0.5 |
| SC4 | *Enterococcus faecalis* strain, human stool isolate, 2012 | 1 | 0.25 |
| SC5 | *Enterococcus faecium* strain, human stool isolate, 2014 | 1 | 0.25 |
| SC7 | *Enterococcus faecalis* strain, human stool isolate, 2014 | 2 | 1 |
| SC11 | *Enterococcus faecium* strain, human stool isolate, 2017 | 1 | 0.25 |
| SC13 | *Enterococcus faecalis* strain, human stool isolate, 2017 | 2 | 0.25 |
| SC17 | *Enterococcus faecium* strain, human stool isolate, 2017 | 1 | 0.5 |
| SC22 | *Enterococcus faecalis* strain, human stool isolate, 2017 | 1 | 0.25 |
| ATCC700802(V583) | Vancomycin resistant *enterococcus faecalis* strain, purchased from ATCC | 2 | 128 |
| Vr12-1 | Vancomycin resistant *enterococcus faecalis* strain, human stool isolate, 2015 | 1 | 32 |
| Vr12-2 | Vancomycin resistant *enterococcus faecalis* strain, human stool isolate, 2015 | 2 | 64 |
| Vr12-3 | Vancomycin resistant *enterococcus faecalis* strain, human stool isolate, 2015 | 4 | 64 |
| Vr12-4 | Vancomycin resistant *enterococcus faecium* strain, human stool isolate, 2015 | 1 | 32 |
| Vr12-5 | Vancomycin resistant *enterococcus faecalis* strain, human stool isolate, 2015 | 1 | 32 |
| Vr16-4 | Vancomycin resistant *enterococcus faecalis* strain, human stool isolate, 2015 | 2 | 16 |
| Vr16-7 | Vancomycin resistant *enterococcus faecium* strain, human stool isolate, 2017 | 2 | 64 |
| Vr18-1 | Vancomycin resistant *enterococcus faecalis* strain, human stool isolate, 2017 | 1 | 64 |
| Vr18-2 | Vancomycin resistant *enterococcus faecalis* strain, human stool isolate, 2017 | 1 | 128 |

Table S1. The MIC of CSA and vancomycin inhibited *enterococcus faecalis.*

Table S2. Gene name and Primers used in this study.

| **Gene** | **Primer direction** | **Primer sequence** |
| --- | --- | --- |
| *EF_0694* | Sense | 5' GCCGTTTGTAGTAGCAGG 3' |
|  | Antisense | 5' CATAGCAGCGGTAGCATT 3' |
| *EF_0695* | Sense | 5' AGCGAGAACAAGAAGGAA 3' |
|  | Antisense | 5' ACTCTGCCACTCAACACC 3' |
| *EF_3138* | Sense | 5' ATGACGGAAACAACAGAA 3' |
|  | Antisense | 5' AAAATGCCAACGGAGATAGGCTTTT 3' |
| *EF_3307* | Sense | 5' CCAAACAGGAGCAGAAAC 3' |
|  | Antisense | 5' AGGCAGCACTAAATACCG 3' |
| *EF_3305* | Sense | 5' GATGCACCAGATGCTTTA 3' |
|  | Antisense | 5' TACCTCGCCTACAGCGGTAATCTGG 3' |
| *EF_2598* | Sense | 5' TTTTAGCGGTTGCTTTGG 3' |
|  | Antisense | 5' GCGTTGTATCCTTGTCCC 3' |
| *EF_3211* | Sense | 5' GTAGCCGTAGTTTGGACC 3' |
|  | Antisense | 5' AAGACATCAATCGCATCA 3' |
| *EF_0461* | Sense | 5' CCTTGTAATGTGGCTATG 3' |
|  | Antisense | 5' TCACCGCAGATTGGCCAATTTGCGT 3' |
| *EF_3137* | Sense | 5' AAGCAGCCGTCTCACCT 3' |
|  | Antisense | 5' GCCACCACTTCATCGACAACGAAAA 3' |
| *EF_0019* | Sense | 5' CAGGCAGTCGGACAGGA 3' |
|  | Antisense | 5' AAGGCGTTGCTTGGAAATAAATATC 3' |

Table S3. Changes of the expression of all PTS proteins after CSA treatment.

| Protein accession | Protein description | C_CSA/C Ratio | P value |
| --- | --- | --- | --- |
| Q837Y1 | PTS system, fructose-specific family, IIBC components | 0.139 | 0.000036 |
| Q837Y0 | PTS system, IIA component OS=Enterococcus faecalis | 0.154 | 0.000037 |
| Q82ZC6 | PTS system, IID component OS=Enterococcus faecalis | 0.205 | 0.000016 |
| Q82YX4 | PTS system, sorbitol-specific IIC component | 0.272 | 0.000042 |
| Q82YX6 | PTS system, sorbitol-specific IIA component | 0.364 | 0.000001 |
| Q831B4 | PTS system, beta-glucoside-specific IIABC component | 0.379 | 0.000000 |
| Q82Z62 | PTS system, IIB component | 0.394 | 0.000038 |
| Q838I6 | PTS system, IIA component | 0.404 | 0.000004 |
| Q82ZC7 | PTS system, IIB component | 0.492 | 0.000005 |
| Q839Y0 | PTS system, IIB component | 0.496 | 0.000042 |
| Q838J0 | PTS system, IIB component | 0.546 | 0.000003 |
| Q834P2 | PTS system, IIABC components | 0.562 | 0.000121 |
| Q836Y6 | PTS system, IIABC components | 0.674 | 0.000737 |
| Q839X7 | PTS system, mannose-specific IID component | 0.682 | 0.000016 |
| Q839X9 | PTS system, mannose-specific IIAB components | 0.689 | 0.000060 |
| Q837W1 | PTS system, fructose-specific family, IIABC components | 0.697 | 0.000020 |
| Q82ZM2 | PTS system, IIB component | 0.700 | 0.009600 |
| Q834W2 | PTS system, IIABC components | 0.751 | 0.000016 |
| Q836T9 | PTS system, IIA component | 0.76 | 0.000199 |
| Q833X1 | PTS system, IIB component | 0.825 | 0.000278 |
| P23530 | Phosphoenolpyruvate-protein phosphotransferase | 0.833 | 0.000820 |
| Q839X8 | PTS system, mannose-specific IIC component | 0.854 | 0.000179 |
| Q82ZM4 | PTS system, IID component | 0.891 | 0.227260 |
| Q831R5 | PTS system, IIBC components | 1.024 | 0.410000 |
| Q836U5 | PTS system, IIB component | 1.07 | 0.022103 |
| Q832L3 | PTS system, IIBC components | 1.093 | 0.054756 |
| Q836U0 | PTS system, IIB component | 1.261 | 0.014117 |
| Q831B0 | PTS system, IIA component | 1.296 | 0.000220 |
| H7C7A3 | PTS system, IIB component | 1.579 | 0.000043 |
| Q831R2 | PTS system, IIA component | 1.822 | 0.000000 |
